# Comparison of Clinical, Magnetic Resonance Imaging (MRI) and Arthroscopic Findings in Assessment of Cartilage Defects and Internal Derangement of Knee

**DOI:** 10.7759/cureus.40110

**Published:** 2023-06-08

**Authors:** Nandini Sanjay, Arun H Shanthappa, Ajay Kurahatti, Arun Kumaar

**Affiliations:** 1 Department of Orthopaedics, Sri Devaraj Urs Academy of Higher Education and Research, Kolar, IND

**Keywords:** meniscus, arthroscopy, magnetic resonance imaging, clinical evaluation, anterior cruciate ligament, comparison, knee

## Abstract

Background: The knee is the most commonly injured joint because of its anatomical structure, its exposure to external forces, and its functional demands. Orthopaedic surgeons previously relied on clinical evaluation for diagnosing any internal derangement of the knee joint. With the advent of new clinical methods for diagnosing ligament injuries and cartilage defects, there are very less studies comparing the accuracy of all three methods, clinical examination, magnetic resonance imaging (MRI) and arthroscopy to reach a definitive diagnosis.

Objective: This study aims to compare the sensitivity, specificity, accuracy and predictive values of clinical examination and MRI with that of arthroscopy which is the ideal investigation of choice for cartilage defects and internal derangements of the knee.

Material and methods: A prospective, observational and hospital-based study was done on patients with internal derangement of knee and cartilage defects. Clinical examination (based on the clinical tests for each ligament), MRI (1.5 T) and arthroscopy were done on all patients, the findings of which were compared using the Chi-square test. The following parameters were assessed while using arthroscopy as the gold standard of reference: accuracy, specificity, sensitivity, positive predictive value (PPV), and negative predictive value (NPV).

Results: Anterior cruciate ligament (ACL) was the most common ligament to be injured followed by the medial meniscus. The overall accuracy of clinical evaluation and MRI to diagnose meniscal injuries was found to be 94% and 91% respectively. The clinical examination had sensitivity and specificity of 96% and 82% in diagnosing ACL tears, respectively, whereas MRI had sensitivity and specificity of 88% and 76% respectively. For the medial meniscus, the clinical examination had sensitivity and specificity of 93% and 96% respectively whereas MRI had a sensitivity of 100% and specificity of 89%. We observed that the accuracy of MRI for grading ACL and meniscal tears was similar i.e. 79% and 78% respectively, but was slightly low (70%) for grading of chondromalacia patellae.

Conclusion: This study supports the use of MRI and clinical assessment in the diagnosis of chondral defects and internal knee derangement. Clinical tests are reliable and have high sensitivity in diagnosing ACL tears and chondral defects when compared to MRI. Not all lesions should routinely undergo MRI for diagnostic purposes; only a few circumstances warrant its usage. MRI is less reliable in grading ACL tears, meniscal tears and chondral injuries.

## Introduction

The knee is the most commonly injured joint because of its anatomical structure, its exposure to external forces, and its functional demands. [1,2] Ligament injuries are among the most frequent knee injuries resulting from contact sports like hockey, football, and kabaddi, however, RTAs and workplace accidents also contribute.

The most common ligament to be injured, causing knee instability and pain, is the anterior cruciate ligament (ACL) [3,4]. Meniscus tears can be brought on by trauma or develop over time as a result of the instability brought on by ACL damage. Meniscal and cartilage lesions in the knee joint are frequently accompanied by long-standing ACL injuries [5].

Even for skilled surgeons, it can be challenging to make a proper preoperative diagnosis of a knee internal derangement, which makes it possible for ligament injuries, meniscal, and cartilage defects to be misdiagnosed.

The extent of knee damage can be evaluated clinically, but the development of magnetic resonance imaging (MRI) allows for a more precise diagnosis of soft tissue and cartilage lesions in the knee. In the assessment of knee injuries, MRI has proven to be a quick and non-invasive imaging alternative to physical examination. However, due to the presence of hemarthrosis, pain, and limited range of motion, diagnostic accuracy is low, particularly in patients with acute ACL injury. Clinical findings and MRI interpretation for the above patients can be challenging.

MRI is currently the non-invasive investigation of choice in planning the management of meniscal and ligament injuries.

There are some disagreements over the correlation of MRI and clinical diagnosis with knee joint arthroscopy results [6,7]. According to literature reports, the accuracy of clinical examination of the knee to diagnose meniscal injury is about 64-85% and is about 90-100% for ACL injury [8].

MRI helps in assessing occult bone contusion and soft tissue trauma like ligament and meniscal tears [9]. Eighty-five per cent of meniscal tears and 90% to 100% of ACL injuries are correctly identified by MRI [10], although the sensitivity of the scanner affects the accuracy of the diagnosis [11].

This study's objective was to assess the efficacy of clinical assessment, MRI, and arthroscopy findings in knee injuries, with the latter serving as the gold standard.

## Materials and methods

Forty-three patients with suspected knee injuries presenting to a tertiary care hospital were taken up for the study.

After obtaining informed consent from the patients who agreed to be part of the study, demographic data, history, clinical examination during admission, radiographs of the knee joint to rule out any fractures and all intra-articular findings with regard to knee derangement on MRI were documented in the study proforma, and were taken up for arthroscopy. For future reference, videos and pictures were captured and stored.

A systematic objective examination was conducted before the MRI evaluation. Knee objective evaluation using joint line tenderness, meniscal clinical tests, McMurray test and signs of ligament laxity (Lachman test, anterior and posterior drawer tests, varus and valgus stress tests) were elicited. MRI images were carefully reviewed for signs of soft tissue injuries near the knee joint, as well as damage to the cruciate ligaments, collateral ligaments, menisci, meniscal cysts, loose bodies, articular cartilage, and bone contusions. Diagnostic and therapeutic arthroscopy was done for all patients, findings of which were separately noted for all 43 patients and necessary repair/reconstruction of the ligament/cartilage was done.

The inclusion criteria for our study were patients between 18 and 50 years of age presenting with suspected knee injuries. The exclusion criteria were patients with associated fractures around the knee joint, previous history of knee surgery, degenerative knee joint disorders, isolated collateral ligament injuries, infectious and inflammatory conditions of the knee joint, ferromagnetic implants, pacemakers and aneurysm clips.

Clinical Examination - Anterior and Posterior Drawer Tests were graded as follows: grade I: 2-5 mm; grade II: 5-10 mm; grade III: more than 10 mm.

MRI - meniscal tears were graded into three types as depicted below (Table 1) and chondromalacia patella was graded as per the modified Outerbridge classification (Table 2).

**Table 1:** Depicting grades of meniscal injury (T2 weighted and proton density images)

Grade	Description
1	Small area of hyper intensity, no extension to the articular surface
2	Linear areas of hyper intensity, no extension to the articular surface
2A	Linear abnormal hyper intensity with no extension to the articular surface
2B	Abnormal hyper intensity, reaches the articular surface on single image
2C	Globular wedge-shaped abnormal hyper intensity with no extension to the articular surface
3	Abnormal hyper intensity extends to at least one articular surface (superior or inferior) and is referred to as a definite meniscal tear

**Table 2:** Modified Outerbridge classification MRI: magnetic resonance imaging

Grade	MRI	Arthroscopy
I	Focal areas of hyper-intensity with normal contour	Softening of cartilage, easily indented with probe
II	Blister-like swelling or fraying of articular cartilage extending to the surface	Surface fibrillation
III	Partial thickness cartilage loss with focal ulceration, ‘crab meat’ appearance	Full thickness fissuring or splitting of cartilage
IV	Full thickness cartilage loss with underlying bone reactive changes	Complete loss of cartilage with exposed subchondral bone

Statistical analysis

Collected data were entered in Microsoft Excel (Microsoft®, Redmond, WA, USA) and analyzed using the Statistical Package for the Social Sciences version 20 (IBM, Armonk, NY, USA). For medial meniscus (MM), lateral meniscus (LM), anterior and posterior cruciate ligaments, and cartilage surfaces, statistical analysis was carried out using arthroscopy as the "gold standard." Clinical examination and MRI results were analysed with the Chi-square test. Sensitivity, specificity, accuracy, positive predictive value (PPV) and negative predictive value (NPV) were evaluated considering arthroscopy as the standard of reference. The study required investigations to be done on human subjects mandatory for anaesthesia, arthroscopy and MRI of the knee joint. Surgical intervention was undertaken after adequate preoperative assessment and after taking informed written consent.

## Results

The average age in our study population was 30 years. The youngest patient being 19 years and the oldest patient being 50 years. Most of the patients were within the age group of 19-25 years. In this study, 11 cases were female and 32 cases were male. Male to female ratio for the whole series was 3:1. There were 22 patients with left knee involvement and 21 patients with right knee. No bilateral knee cases were present. Twenty-seven patients (62.8%) sustained twisting injuries of the knee. Only three patients (7%) sustained direct trauma to the knee.

**Figure 1:**
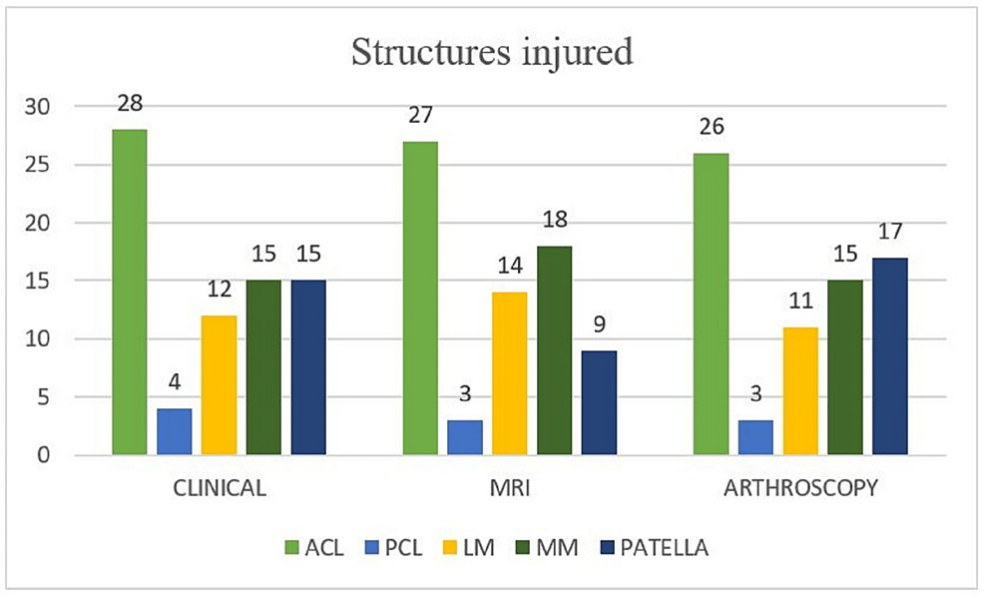
Distribution of structures injured among study participants y-axis - number of patients MRI - magnetic resonance imaging; ACL - anterior cruciate ligament; PCL - posterior cruciate ligament; LM - lateral meniscus; MM - medial meniscus

We observed that the ACL was most commonly injured among the study population followed by MM and patellar cartilage (Figure 1).

ACL injury

In the table below (Table 3), we have compared the findings of ACL injury on clinical examination and arthroscopic evaluation. We observed that 25 patients had positive clinical and arthroscopic findings i.e. tear was clinically suspected after performing the tests for ACL injury, the same was confirmed on arthroscopy. The remaining four patients showed a discrepancy between clinical examination and arthroscopic findings. It was observed that, with respect to ACL injury, clinical examination has a sensitivity of 96.1% and specificity of 82.3%. The accuracy of clinical examination to detect ACL tears in the study population was observed to be 90%.

**Table 3:** Cross-tabulation between clinical findings and arthroscopy (gold standard) for ACL injury ACL - anterior cruciate ligament

ACL		Arthroscopy Tear	Arthroscopy Normal	Total
Clinical finding	Positive	25 (58.1%)	3 (7.0%)	28 (65.1%)
	Negative	1 (2.3%)	14 (32.6%)	15 (34.9%)
	Total	26 (60.5%)	17 (39.5%)	43 (100%)

**Figure 2:**
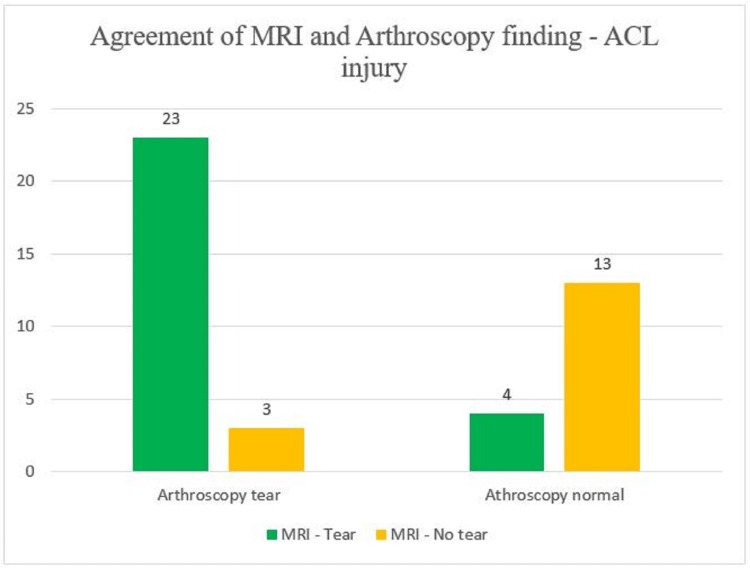
Comparison of MRI and arthroscopy finding (gold standard) for ACL injury y-axis - number of patients MRI - magnetic resonance imaging; ACL - anterior cruciate ligament

With regard to MRI findings, we observed that 23 patients had positive MRI and arthroscopic findings that is, an ACL tear was present on MRI evaluation of the affected knee joint and the same was correlated on arthroscopy. The remaining seven patients showed a discrepancy in MRI and arthroscopic findings (Figure 2).

After comparing MRI and arthroscopic findings of ACL injury, it was noted that MRI has a sensitivity of 88% and specificity of 76.4%. The accuracy of MRI to detect ACL tears in the study population was observed to be 83.7%.

Posterior cruciate ligament (PCL) injury

We observed that three patients had positive clinical and arthroscopic findings, which implied, the positive result of the clinical test led to suspicion of PCL tear which was correlated with arthroscopy. One patient showed a discrepancy between clinical examination and arthroscopic findings.

With respect to PCL injury, it was ascertained that clinical examination has a sensitivity of 100% and specificity of 97.5%. The accuracy of clinical evaluation to detect PCL tears in the study population was noted to be 97.7%.

On comparing MRI and arthroscopic findings, we observed that three patients had positive MRI and arthroscopic findings which implied PCL tear was present on both MRI evaluation and arthroscopy. The remaining 40 patients showed no tears on MRI and on arthroscopy.

For a total number of 43 patients that were studied, we found that sensitivity, specificity and accuracy by MRI evaluation of PCL tear is 100%.

LM injury

We observed that 10 patients had positive clinical and arthroscopic findings, which implied, the positive result of the clinical test led to the suspicion of LM tear which was confirmed on arthroscopy. Three patients showed discrepancies in clinical examination and arthroscopic findings.

For LM injury, we observed that clinical examination has a sensitivity of 90.9% and a specificity of 93.7%. The overall accuracy of clinical evaluation to detect LM tears in the study population was noted to be 93%. Ten patients had positive MRI and arthroscopic findings which implied LM tear was present on both MRI evaluation as well as on arthroscopic evaluation. Five patients showed a discrepancy in MRI and arthroscopic findings. In the study population, it was observed that MRI has a sensitivity of 90.9% and specificity of 87.5% in diagnosing LM tears and an accuracy of 88.4%.

MM injury

In the table below (Table 4), a comparison of findings of MM injury on clinical examination and arthroscopic evaluation has been documented. It was observed that 14 patients had positive clinical and arthroscopic findings i.e. tear was clinically suspected after performing the tests for MM injury, the same was confirmed on arthroscopy. Two patients showed a discrepancy between clinical examination and arthroscopic findings.

**Table 4:** Cross-tabulation between clinical findings and arthroscopy (gold standard) for medial meniscus injury

Medial Meniscus		Arthroscopy Tear	Arthroscopy Normal	Total
Clinical finding	Positive	14 (32.6%)	1 (2.3%)	15 (34.9%)
	Negative	1 (2.3%)	27 (62.8%)	28 (65.1%)
	Total	15 (34.9%)	28 (65.1%)	43 (100%)

It was observed that, with respect to MM injury, the clinical examination has a sensitivity of 93.3% and a specificity of 96.4%. The overall accuracy of clinical examination to detect MM tears in the study population was observed to be 95.3%.

**Figure 3:**
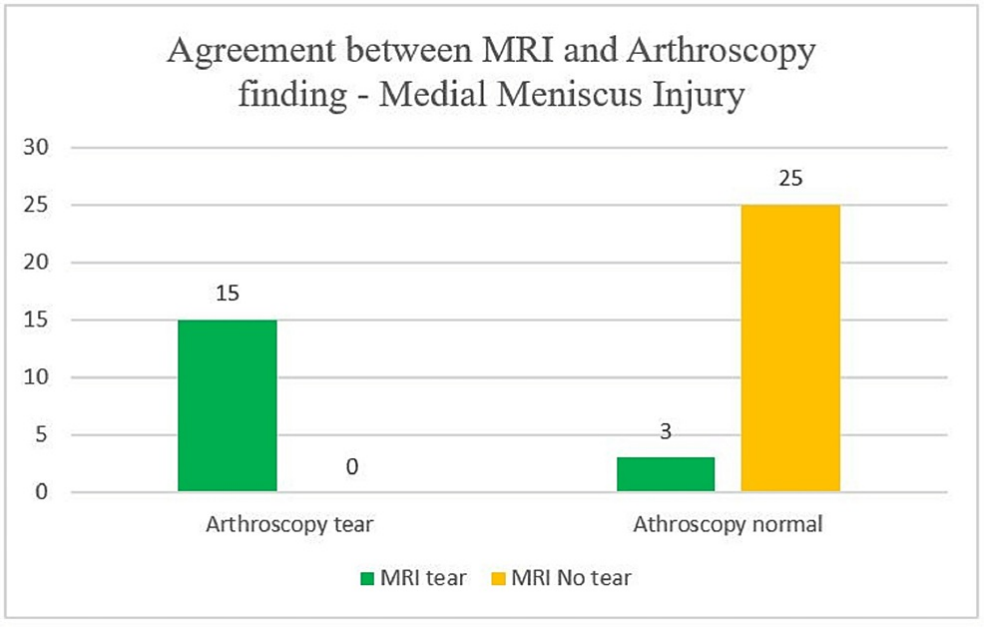
Comparison between MRI and arthroscopy findings (gold standard) for medial meniscus injury y-axis - number of patients MRI - magnetic resonance imaging

With regard to MRI findings, we observed that 15 patients had positive MRI and arthroscopic findings i.e. MM tear was present on MRI evaluation of the affected knee joint and the same was confirmed on arthroscopy. Three patients showed a discrepancy between MRI and arthroscopic findings (Figure 3).

After comparing MRI and arthroscopic findings of MM injury, it was noted that MRI has a sensitivity of 100% and a specificity of 89.2%. The overall accuracy of MRI to detect MM tears in the study population was observed to be 93%.

Patellar articular cartilage

In the below table (Table 5), clinical examination and arthroscopic evaluation of patellar cartilage are documented. It shows that 15 patients had positive clinical and arthroscopic findings, which implied, clinical tests led to suspicion of patellar articular damage which was correlated with arthroscopy. Two patients showed a discrepancy between clinical findings and arthroscopic findings.

**Table 5:** Cross-tabulation between clinical findings and arthroscopy (gold standard) for patellar injury

Patella		Arthroscopy Chondromalacia	Arthroscopy Normal	Total
Clinical finding	Positive	15 (34.9%)	0	15 (34.9%)
	Negative	2 (4.6%)	26 (60.5%)	28 (65.1%)
	Total	17 (39.5%)	26 (60.5%)	43 (100%)

We observed that clinical examination has a sensitivity of 88.2%, a specificity of 100% and an accuracy of 95.3% in diagnosing patellar cartilage damage.

**Figure 4:**
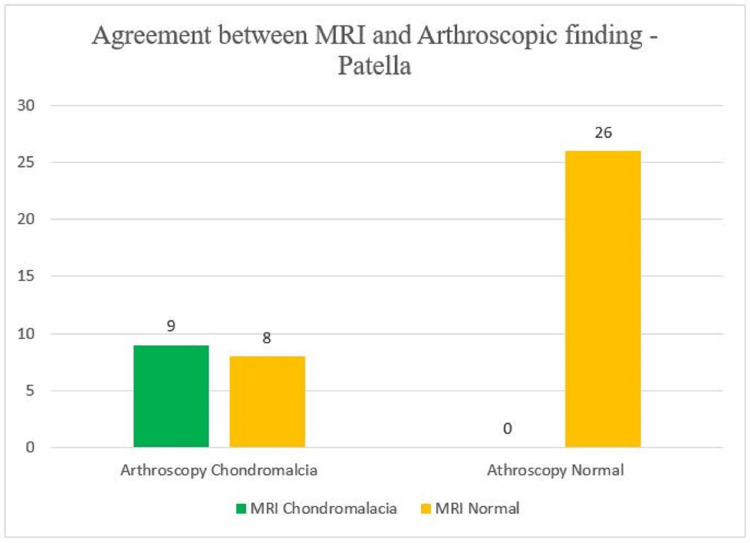
Comparison between MRI and arthroscopic finding (gold standard) for patellar injury y-axis - number of patients MRI - magnetic resonance imaging

With regard to MRI findings, we observed that nine patients had positive MRI and arthroscopic findings that is chondromalacia patella was present on MRI evaluation of the affected knee joint and the same was correlated on arthroscopy. Eight patients showed a discrepancy between MRI and arthroscopy findings (Figure 4).

To detect chondromalacia patella, we observed in our study that MRI has a sensitivity of 53%, specificity of 100% and an accuracy of 81.4%.

On comparing MRI and arthroscopic grades for ACL and meniscal tears we observed that MRI has an accuracy of 79% and 76.7% respectively. For chondromalacia patella, on comparing MRI accuracy with arthroscopic grades it was observed that MRI was 69.8% accurate in the grading of chondromalacia patella.

The tables below summarize the overall sensitivity, specificity and accuracy of clinical examination and MRI in internal derangement of the knee and chondromalacia patella (Tables 6, 7, 8).

**Table 6:** Sensitivity, specificity and accuracy of clinical examination ACL - anterior cruciate ligament; PCL - posterior cruciate ligament; LM - lateral meniscus; MM - medial meniscus

Structure	Sensitivity (%)	Specificity (%)	Accuracy (%)
ACL	96.1	82.3	90
PCL	100	97.5	97.7
LM	90.9	93.7	93
MM	93.3	96.4	95.3
Patella cartilage	88.2	100	95.3

**Table 7:** Sensitivity, specificity and accuracy of magnetic resonance imaging (MRI) ACL - anterior cruciate ligament; PCL - posterior cruciate ligament; LM - lateral meniscus; MM - medial meniscus

Structure	Sensitivity (%)	Specificity (%)	Accuracy (%)
ACL	88	76.4	83.7
PCL	100	100	100
LM	90.9	87.5	88.4
MM	100	89.2	93
Patella cartilage	53	100	81.4

**Table 8:** Accuracy of MRI grading ACL - anterior cruciate ligament; MRI: magnetic resonance imaging

Structure	Accuracy (%)
ACL	79
Meniscus	76.7
Patella cartilage	69.8

In our study, we found that MRI had higher false positives i.e. high sensitivity and low specificity (Table 9) which implies that if MRI is used as the form of pre-operative screening for the condition, then there can be unnecessary arthroscopies performed, which is contradictory to studies saying that MRI prevents unnecessary arthroscopy [12].

**Table 9:** False positives in MRI ACL - anterior cruciate ligament; LM - lateral meniscus; MM - medial meniscus; MRI: magnetic resonance imaging

Structures	False Positive Patients
ACL	4
LM	4
MM	3

## Discussion

The primary goal of our study is to compare the diagnostic precision of clinical examination, MRI findings, and arthroscopy in the detection of cartilage defects and internal knee derangements.

Men experience knee injuries more frequently than women do, and the right knee is more commonly injured than the left, according to Nageswara et al. [13]. This finding was similar to our study's findings, which revealed that men were more frequently affected but the left knee was more frequently involved.

Uppin et al. [14] conducted a study in which the maximum number of patients were between 20 and 40 years and the most common cause of knee injury was RTA followed by sports injuries. When compared to our study, we also observed that the maximum number of patients were between 19 and 40 years of age and the most common cause of injury was observed to be a twisting force.

According to our findings, MM injuries occurred more frequently than LM injuries.

We observed that the majority of patients had isolated cruciate ligament injury (39.5%), followed by combined cruciate and meniscal injury (27.9%).

We noted that 17 patients had chondromalacia patella. We also observed that chondromalacia was present in six patients with ACL injuries and in three patients with meniscal tears.

Oksman et al. [15] conducted a study which showed 28.8% of patients had chondromalacia patella in ACL-deficient knees, the same of which was comparable to our study which showed 35.2% of patients had chondromalacia patella in ACL deficient knees.

The present study showed 17.6% of patients had meniscal tears associated with chondromalacia patella whereas, in a study conducted by Resorlu et al. [16] the authors observed 38% of patients who had meniscal injury also had chondromalacia patella.

Some authors also proposed that clinical examination and MRI evaluation show no significant differences in the diagnostic accuracy of ACL and meniscal injuries [17,18].

Kocabey et al. [19] concluded that there are no real advantages of MRI over clinical examination in the diagnosis of meniscal and ACL tears before knee arthroscopy.

Orlando et al. [20] in their prospective study on 72 patients showed that clinical examination had a sensitivity, specificity and accuracy of 88.67%, 94.73% and 90.27% respectively, in diagnosing ACL injuries. The same can be comparable with this study which showed sensitivity, specificity and accuracy of 96.1%, 82.3% and 90% respectively, in diagnosing ACL tears.

Nageswara et al. [13] conducted a cross-sectional study, which showed clinical examination had a sensitivity of 62% and specificity of 48% to diagnose medial meniscal tears. For LM tears, clinical examination showed a sensitivity of 58% and specificity of 54%. The values greatly vary when compared to the present study which showed clinical examination has sensitivity and specificity of 93.3% and 96.4% respectively, for diagnosing MM tears. For diagnosing LM tears, clinical examination showed sensitivity and specificity of 90.9% and 93.7% in the present study.

Shahani et al. [21] conducted a study which showed clinical examination findings were having a higher sensitivity to diagnose MM and ACL tears while it shows low sensitivity to LM tears. A similar observation was made in the present study. The authors also observed that while specificity for a clinical examination to diagnose LM and ACL tears was high, it was low for MM tears. In contrast to the above, our study showed high specificity for meniscal tears (both, medial and lateral) but low specificity for ACL tears.

MRI has shown to be reliable, safe, and beneficial compared to diagnostic arthroscopy, which is now referred to as the standard reference for diagnosing knee problems. Arthroscopy is an invasive procedure that entails anaesthetic and surgery risks, including neurovascular injury, infection, and postoperative pain. If other non-invasive diagnostic imaging modalities, such as MRI, are available, it is preferable to use them for therapeutic purposes [22].

Elvenes et al. [23] found that the PPV, NPV, specificity and sensitivity of MRI were 71%, 100%, 77% and 100% respectively for MM tears.

Polly DW et al. [24] in a comparative study of 54 patients with selective MRI and arthroscopy found sensitivity, specificity and accuracy of 95.8%, 100% and 98% respectively for MM tear, 66.7%, 95.1% and 90% for LM tear, 100%, 96.9% and 97.3% for ACL injuries.

We observed that PPV, NPV, sensitivity and specificity of MRI to diagnose MM injuries were 83.3%, 100%, 100% and 89.2% respectively, which correlates with the findings of the above-mentioned studies.

We also observed that MRI had a substantial agreement with arthroscopy in diagnosing LM tears (accuracy of 88.4%) when compared to an almost perfect agreement for MM tears (accuracy of 93%).

In the current study, sensitivity and specificity values for MRI compared with arthroscopy are 88% and 76.4% respectively, for ACL injuries.

We found that clinical examination had better sensitivity, specificity, predictive values and diagnostic accuracy in comparison to MRI to diagnose ACL tears which were in congruence with a cross-sectional study done by Rayan et al. [25] who concluded that carefully performed clinical examination can give equal or better diagnosis of meniscal and ACL injuries in comparison to MRI scan and recommended that MRI be used to rule out such injuries rather than to diagnose them.

In the present study, three cases of PCL tear had been detected both by MRI and arthroscopy, which showed 100% sensitivity, specificity and accuracy.

The table below (Table 10) shows the results of the comparison of clinical findings and arthroscopy findings for ACL tears in our study and different studies.

**Table 10:** The results of clinical and arthroscopy findings for ACL tears of our study compared to other studies PPV - positive predictive value; NPV - negative predictive value; ACL - anterior cruciate ligament

Name of Study	Sensitivity (%)	Specificity (%)	PPV (%)	NPV (%)	Accuracy (%)
Dutka et al. [26]	86	90	94	79	88
Rayan et al. [25]	77	100	100	95	96
Navali et al. [27]	99	92	95	98	96
Present Study	96	82	89	93	90

The results of the comparison between MRI and arthroscopy findings for ACL tears in our study and different studies are shown below (Table 11).

**Table 11:** The results between MRI and arthroscopy findings for ACL tears of our study compared to others PPV - positive predictive value; NPV - negative predictive value; ACL - anterior cruciate ligament

Name of Study	Sensitivity (%)	Specificity (%)	PPV (%)	NPV (%)	Accuracy (%)
Dutka et al. [26]	80	86	90	72	82
Rayan et al. [25]	81	96	81	95	93
Navali et al. [27]	99	83	90	98	93
Present Study	88	76	85	81	84

Results obtained from the current study were similar to or equal to the results of other studies after comparing clinical, MRI and arthroscopic findings for ACL tears.

Results obtained by our study were better than or similar to the results of other studies after comparing clinical, MRI and arthroscopy findings for LM tears.

Results obtained by our study were better than or similar to the results of other studies after comparing clinical, MRI and arthroscopy findings for MM tears.

In the current study, we observed that MRI altogether has a low PPV meaning it was less likely for patients who had a tear in MRI, to have the same observation in arthroscopy when compared with clinical examination.

With regard to the detection of chondromalacia patellae, according to a study conducted by Harri K et al. [28], the PPV of MRI was 75%, the NPV was 72%, the sensitivity was 60%, the specificity was 84%, and the diagnostic accuracy was 73%. When compared to the present study data, it was noted that the results obtained were similar to the above-mentioned study. We noted a PPV of 100%, NPV of 76%, sensitivity was 53%, specificity was 100% and diagnostic accuracy was 81%.

Mattila et al. [29] conducted a study to detect the sensitivity of MRI for diagnosing chondromalacia patellae and observed that 64% of patients had grade I articular cartilage lesions of the patella on arthroscopy which were not visible in MRI. In comparison with our study, we observed that 75% of patients had grade I chondromalacia patellae which were not visible on MRI. The same was seen by Heron et al. [30] in a study that showed MRI can satisfactorily detect II and III-grade chondral defects other than the damages at the patellar articular cartilage, but is unreliable for grade I cartilage defects.

In the present study, we observed that the accuracy of MRI for grading ACL and meniscal tears is similar that is 79% and 78% respectively but was slightly low (70%) for grading of chondromalacia patellae.

Limitations

In our study, clinical evaluation and MRI demonstrate less specificity to identify the knee's internal derangement as compared to arthroscopy. This can be due to the study's limitations, which include the fact that it is a prospective, non-randomized study with a small sample size. Since PCL injury was seen only in three patients, the results obtained cannot be implied to be significant. Medial collateral and lateral collateral ligament injuries were rarely seen in the study population hence; it has not been included in statistical analysis. Our study has not included the time at which MRI and arthroscopy should be done to reduce the number of false positive cases. We have also not considered inter-observer variation in terms of reporting the MRIs performed.

## Conclusions

We observed that MRI is a non-invasive investigation having moderate sensitivity, specificity and accuracy to diagnose meniscal and ACL injuries. This study refuted claims made by other studies that MRI was more accurate than arthroscopy in identifying meniscal and ACL injuries.

In our study, we observed that the accuracy of clinical examination was higher in meniscal and ACL tears when compared to MRI, which showed fairly low accuracy to diagnose tears of the above-mentioned structures.

According to the literature, MRI is 90% accurate at detecting ACL and MM injuries is significantly less for diagnosing LM tears unlike in the present study, where, the accuracy of MRI is low in diagnosing ACL and LM tears when compared to the accuracy of MRI to diagnose MM tears, which was significantly high.

With reference to chondral defects, in agreement with most of the studies, MRI has low accuracy but excellent specificity. It was also observed that it is less sensitive to detect ACL injuries and chondral defects.

Following diagnostic arthroscopy, we observed that inferior surface meniscal tears are sometimes difficult to identify, hence MRI can play a major role in diagnosing these tears.

The effect of these results can greatly improve procedural time by planning with more specificity and thereby reducing surgical risks and unnecessary financial burden on the patient.

## Figures and Tables

**Figure 5:**
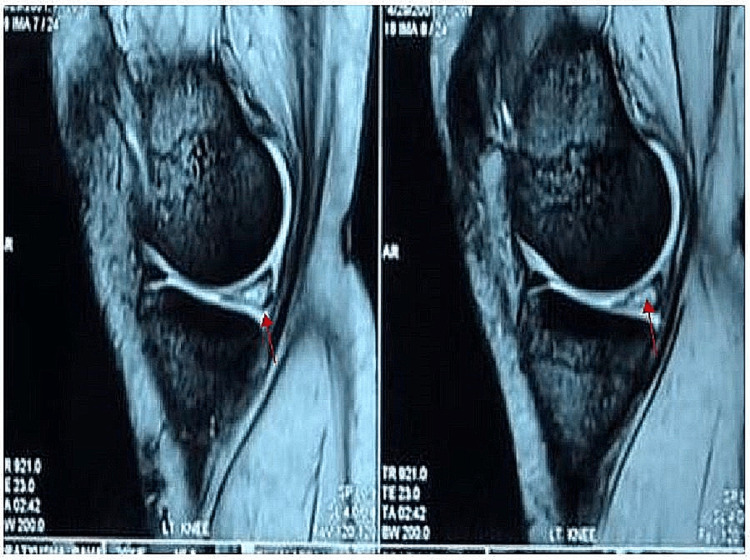
Sagittal MRI findings of the posterior horn of medial meniscus tear (red arrows) MRI: magnetic resonance imaging

**Figure 6:**
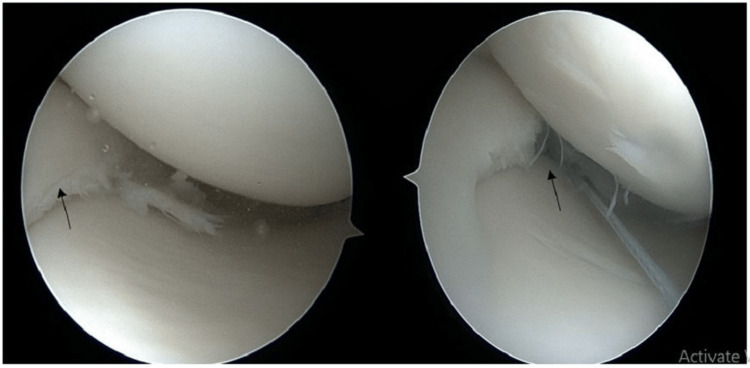
Arthroscopic finding of the above patient showing posterior horn tear (black arrows)

**Figure 7:**
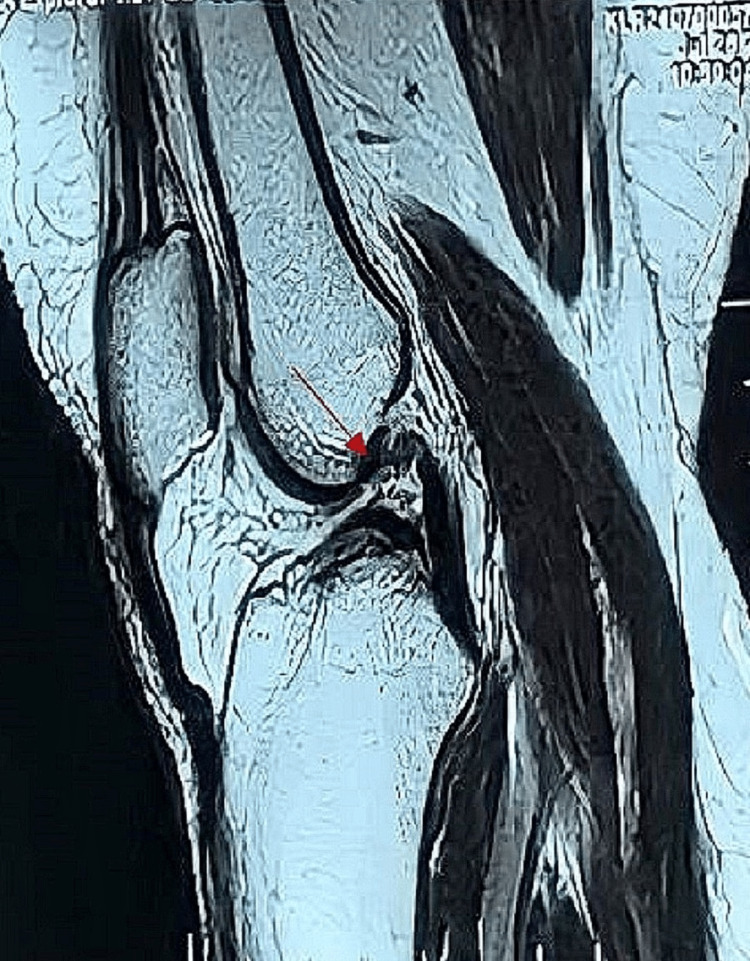
Sagittal MRI image of partial ACL tear (red arrow) MRI - magnetic resonance imaging; ACL - anterior cruciate ligament

**Figure 8:**
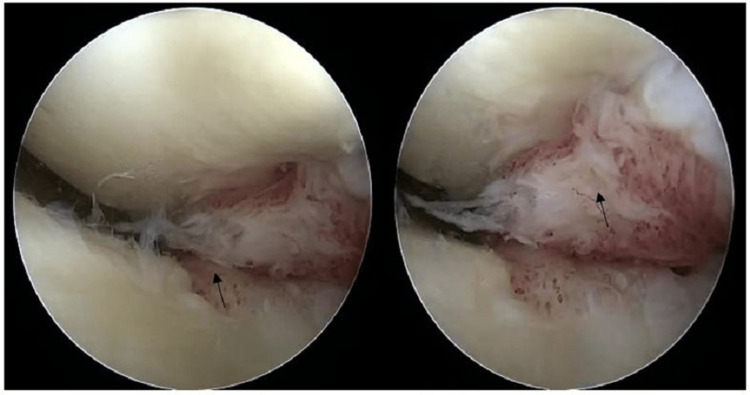
Arthroscopic image of the above patient showing complete ACL tear (black arrows) ACL - anterior cruciate ligament

## Appendices

Depicted below are the MRI and arthroscopic images of two cases included in the study.

Case 1 - (Figures 5, 6)

Clinical examination: McMurray’s positive for MM

MRI: Tear in posterior horn of MM

Arthroscopy: MM tear

Case 2 - (Figures 7, 8)

Clinical examination: Anterior drawer test positive (Grade 2)

MRI: Partial ACL tear

Arthroscopy: Complete ACL tear
